# Translation and Cross-Cultural Adaptation of an Arabic Version of the Summated Xerostomia Inventory

**DOI:** 10.7759/cureus.47546

**Published:** 2023-10-23

**Authors:** Saleh Alqaryan, Hisham Almousa, Saif Almeshari, Mashal B Abaalkhail, Abdulaziz M Alabdulkareem, Shams Alotaibi, Khalid Al-Qahtani

**Affiliations:** 1 Department of Otolaryngology-Head and Neck Surgery, King Saud University, Riyadh, SAU; 2 Department of Medicine, College of Medicine, King Saud University, Riyadh, SAU; 3 Department of Otolaryngology-Head and Neck Surgery, King Fahad Medical City, Riyadh, SAU

**Keywords:** cross-cultural adaptation, translation, sxi questionnaire, dry mouth, xerostomia

## Abstract

Objective: We aim to assess the validity and reliability of the Arabic language translation of the Summated Xerostomia Inventory (SXI).

Methods: A cross-sectional, self-administered, electronic Arabic SXI was sent to 79 patients with thyroid nodules (female: n = 34, 57%; male: n = 45, 57%) who visited the Otolaryngology-Head and Neck Surgery clinic at King Abdulaziz University Hospital and King Fahad Medical City between June 2023 and July 2023.

Results: Dependability was assessed using Cronbach’s alpha coefficients at two distinct instances (first: α = 0.824, second: α = 0.932), which reaffirmed the SXI’s reliability and consistency. Legitimacy was ascertained using a test-retest method and correlation analysis between the two measurements.

Conclusion: All SXI items displayed a potent positive correlation (between 0.746 and 0.871, p < 0.001), exhibiting remarkable consistency in responses over time. The outcomes of the paired t-tests showed nonsignificant differences for all queries, indicating that the responses were stable over time.

## Introduction

Dry mouth, which is a common symptom of being upset, anxious, or stressed, is a subjective sensation of less saliva being secreted in the mouth. Living with less saliva is not only challenging, but it can also result in serious health problems such as xerostomia [1].

Low salivary flow and the subjective experience of dry mouth are both forms of dry mouth; the former is known as salivary gland hypofunction (SGH) and the latter as xerostomia. Low salivary flow is a symptom that the patient experiences, while xerostomia is a sign that the clinician can measure. A recent systematic review of epidemiological studies on dry mouth found that the prevalence of SGH was 20%, and that of xerostomia was 23%, with rates for both conditions being higher in the elderly [2]. The majority of studies that were reviewed were conducted on elderly people [2].

Speaking, chewing, tasting, and swallowing can all be negatively impacted by xerostomia with or without hyposalivation, which has a big impact on oral health-related quality of life [3]. The main contributing factor to xerostomia is thought to be medication. Over 400 drugs have been linked to xerostomia; the most common ones are antidepressants, antihypertensives, and antihistamines [4]. Head and neck radiotherapy, Sjögren’s syndrome, anxiety and depression, systemic sclerosis, sarcoidosis, and Parkinson’s syndrome are some other common causes of xerostomia [5].

When diagnosing xerostomia, it is crucial to conduct an appropriate patient evaluation that includes a comprehensive medical-dental history and diagnostic tests, such as measuring salivary flow [6]. There are currently many options available for treating xerostomia, including systemic and local therapies. Systemic treatments such as cevimeline or pilocarpine may be used in patients whose xerostomia is caused by hyposalivation (with remaining glandular acini), but these medications can have serious negative side effects. The most preferred form of treatment is local therapy, which includes salivary stimulants and salivary substitutes. Local therapies reduce symptoms with little to no negative side effects [7].

The Xerostomia Inventory (XI), which consists of 11 questions to detect and measure the severity of xerostomia, was first described by Thomson et al. in 1999 [8]. Later, a short-form version, the Summated XI (SXI), was established. The SXI is better suited for elderly individuals because it has fewer items and a simpler response format. The SXI questionnaire does not include items from the XI that are unrelated to dry mouth, such as dryness of the eyes, nose, and facial skin. The SXI questionnaire, which has been validated and translated into Dutch, English, Chinese, Portuguese, and Japanese, has been used to measure xerostomia in a number of different countries [9]. Thus, the aim of the present study is to assess the validity and reliability of the Arabic language translation of the Summated Xerostomia Inventory (SXI).

## Materials and methods

This cross-sectional study included all patients who underwent parotid surgery of the head and neck region at the Otolaryngology-Head and Neck Surgery clinic of King Abdulaziz University Hospital and King Fahad Medical City in Riyadh, Saudi Arabia. Patients with systemic dryness-causing diseases such as Sjögren’s syndrome and systemic sclerosis were excluded. The study was approved by the Research Ethics Committee of King Fahad Medical City (institutional review board (IRB) log number: 23-272). Translation, synthesis, back translation, expert committee review, and pretesting were conducted to assess the validity and reliability of the Arabic language translation of the SXI. Between July 2023 and August 2023, a self-administered, electronic Arabic language translation of the SXI was sent to patients who met our inclusion criteria. A retest questionnaire was sent two weeks later to ensure the validity of the responses.

The XI is an 11-item summated rating scale that gives a single continuous scale score representing the severity of chronic xerostomia. The SXI uses five of the 11 items from the XI. We used the SXI to assess the prevalence and severity of xerostomia among patients with head and neck cancers. It is self-explanatory and composed of five items that examine the frequency of dry mouth, difficulty eating dry food, dry mouth when eating a meal, difficulty swallowing certain foods, and dry lips. Each item is scored on a five-point scale (1 = never, 2 = almost never, 3 = sometimes, 4 = often, and 5 = always), with a maximum overall score of 25. A higher rating indicates higher prevalence and severity.

The research was conducted ethically, with all study procedures being performed in accordance with the requirements of the World Medical Association’s Declaration of Helsinki. Verbal informed consent was obtained from each participant/patient for study participation and data publication.

The Shapiro-Wilk test was used to determine the means (M) and standard deviations (SD) of the continuous variables. Categorical variables were summarized as counts and percentages. Cronbach’s alpha coefficient was used to test internal consistency, and values greater than 0.7 were considered acceptable. We tested the validity of each question as well as the total of all five questions using paired-sample t-tests. Reproducibility, or test-retest reliability, was assessed using Pearson’s correlation coefficient (r) between the first and second surveys administered. p-values less than 0.05 (two-sided) were considered statistically significant. Statistical analyses were performed using Statistical Package for the Social Sciences (SPSS) version 26 (IBM Corporation, Armonk, NY).

## Results

Participant characteristics

The Arabic SXI was developed using a survey conducted among a diverse group of participants. This sample encompassed a total of 79 individuals, 43% of whom were female (n = 34) and 57% male (n = 45). The participants were further segmented based on their final pathology classification. A majority (n = 58, 73.4%) were diagnosed with benign conditions, 17.7% (n = 14) had malignant diseases, and the remaining 8.9% (n = 7) were diagnosed with non-neoplastic conditions. In terms of lifestyle habits, a substantial number of participants (n = 68, 86.1%) were nonsmokers, while the rest (n = 11, 13.9%) were smokers. The participants’ ages, height, and weight covered a broad spectrum. Participants’ ages ranged from five to 91 years, with a mean of 40.76 years and a standard deviation of 16.38. The height of participants varied from 1.43 m to 1.84 m. Lastly, participants’ weight ranged from 19 kg to 143 kg, with an average weight of approximately 77.97 kg and a standard deviation of 20.30 kg. In conclusion, the development of the Arabic SXI scale utilized data from a wide variety of individuals, considering factors such as their gender, pathological status, smoking habits, age, height, and weight. This comprehensive collection of participant data ensured the scale’s dependability and versatility across diverse demographic categories (Table 1).

**Table 1:** Demographic characteristics of the participants

Demographic characteristics		Mean	Standard deviation
Age		40.76	16.37
Height		118.95	74.58
Weight		77.97	20.30
Gender		Frequency	Percentage
	Female	34	43%
	Male	45	57%
Type of surgery			
	Partial superficial parotidectomy	5	6.3%
	Radical parotidectomy	2	2.5%
	Superficial parotidectomy	51	64.6%
	Superficial parotidectomy + neck dissection	1	1.3%
	Total parotidectomy	16	20.3%
	Total parotidectomy + neck dissection	4	5.1%
Category of final pathology			
	Benign	58	73.4%
	Malignant	14	17.7%
	Non-neoplastic	7	8.9%
Further treatment			
	Nil	68	86.1%
	Chemotherapy	1	1.3%
	Chemotherapy + radiotherapy	1	1.3%
	Radiotherapy	8	10.1%
	Skin flap	1	1.3%
Smoking			
	No	68	86.1%
	Yes	11	13.9%

Reliability analysis

The reliability of the Arabic SXI was examined using Cronbach’s alpha, a statistical measure often utilized in social sciences and psychology to evaluate a test’s internal consistency or reliability. This is particularly important for multi-item scales, as the goal is to ensure that all items in the scale measure the same latent variable, contributing to the overall score. For the SXI, Cronbach’s alpha values were taken at two different time points. During the first evaluation, a Cronbach’s alpha coefficient value of 0.824 was observed, implying a high level of internal consistency among the five items in the scale. A score above 0.7 is typically considered acceptable, demonstrating that the items have a good degree of internal consistency. For the second evaluation, the Cronbach’s alpha value increased to 0.932. This even superior value further highlights the Arabic SXI scale’s robust internal consistency and dependability. Scores over 0.9 are considered exceptional, indicating the scale’s reliability over time, which is especially important for longitudinal studies or when the tool is used repeatedly. The robustness and dependability of the SXI scale are highlighted by the reliability analysis results, confirming its application for xerostomia measurement. According to the high Cronbach’s alpha values, the scale items are closely related as a whole and contribute to a single unidimensional measure. As a result, the SXI can be regarded as a trustworthy tool in the area it is meant to measure.

Reliability analysis using the test-retest method

The reliability of the five-item measure utilized in this study was also evaluated using the test-retest methodology, adding to the confirmation of the instrument’s consistency over time. The first and second measures were the two time points used in this method to compare the consistency of replies to the same objects. Calculations were made for each item’s mean (M), standard deviation (SD), and correlation coefficient (r) between the results of the two measures (Table 2).

**Table 2:** Descriptive and correlation results of questions for both measures p-value less than 0.05 is considered significant. M: mean, SD: standard deviation, r: correlation coefficient, SXI: Summated Xerostomia Inventory

Question	First measure		Second measure		r	p-value
	M	SD	M	SD		
SXI1	2.28	1.24	2.77	1.19	0.756	<0.001
SXI2	2.04	1.35	2.33	1.40	0.746	<0.001
SXI3	1.82	1.25	2.03	1.30	0.781	<0.001
SXI4	1.78	1.52	2.13	1.31	0.871	<0.001
SXI5	2.48	1.40	2.43	1.33	0.819	<0.001
Standard question	2.58	1.29	2.93	1.17	0.766	<0.001

The test-retest method, which gauges the consistency of responses over time, was also used to evaluate the validity of responses in the SXI. To do this, the correlation between each item on the scale at the two separate time points had to be calculated. A typical validity check question was also supplied. The first item (SXI1) on the scale displayed an average score of 2.28 (SD = 1.24) in the first assessment, which rose to 2.77 (SD = 1.19) in the second evaluation. The connection between the assessments was potent and highly significant (r = 0.756, p < 0.001), signifying stable responses over time. Similarly, the second item (SXI2) recorded an average score of 2.04 (SD = 1.35) for the initial evaluation and 2.33 (SD = 1.40) for the subsequent assessment. Once again, the correlation was potent and highly significant (r = 0.746, p < 0.001), implying high test-retest reliability for this item. The third item (SXI3) marked an average score of 1.82 (SD = 1.25) initially and 2.03 (SD = 1.30) in the following evaluation, accompanied by a significant and potent correlation (r = 0.781, p < 0.001). The fourth item (SXI4) presented the highest correlation of all items (r = 0.871, p < 0.001), suggesting very strong consistency over time. The mean score rose from 1.78 (SD = 1.52) for the first measure to 2.13 (SD = 1.31) for the second measure. The fifth item (SXI5) had almost identical mean scores for the two measures (first: M = 2.48, SD = 1.40; second: M = 2.43, SD = 1.33), indicating stability over time. The correlation was high (r = 0.819, p < 0.001), suggesting strong test-retest reliability. Final evidence supporting the validity of the SXI scale came from the standard question utilized for the validity assessment, which revealed a substantial correlation (r = 0.766, p < 0.001) between the first and second measures (first: M = 2.58, SD = 1.29; second: M = 2.93, SD = 1.17).

In summary, all SXI items and the standard question exhibited high test-retest reliability, as indicated by the strong and significant correlations between the two measurements. These results confirm the reliability of the Arabic SXI, indicating its suitability for repeated measures over time and ensuring its value as a robust tool for assessing xerostomia.

Validity of two measures’ responses

The questionnaire’s validity was assessed by performing paired-sample t-tests on each question and the sum of all five questions. The paired t-test was employed to validate the responses in the Arabic SXI between the two distinct points of measurement. The paired t-test is beneficial for contrasting the average scores of the same group in two separate instances. The first item (SXI1) revealed a mean difference of -0.167 with an SD of 0.834. The t-statistic value was -1.095 with a significance level (p-value) of 0.283. The 95% confidence interval for the mean difference ranged from -0.478 to 0.145, which includes zero, indicating no significant difference in scores over time. Similarly, for the second item (SXI2), the mean difference was -0.233 (SD = 0.971). The calculated t-statistic was -1.316 (p = 0.199). The confidence interval ranged from -0.596 to 0.129, also including zero, implying no significant difference between the two measurements. The third item (SXI3) demonstrated a mean difference of -0.233 (SD = 0.858). The t-statistic was -1.489 (p = 0.147). The confidence interval for this item was from -0.554 to 0.087, again encompassing zero, signifying that the difference between the two measures was not statistically significant. For the fourth item (SXI4), the mean difference was -0.167 (SD = 0.648) with a t-statistic of -1.409 (p = 0.169). The 95% confidence interval was from -0.409 to 0.075, suggesting that there was no significant change in the scores over time. The fifth item (SXI5) displayed a positive mean difference of 0.067 (SD = 0.828). The t-statistic was 0.441 (p = 0.662). The confidence interval for this item ranged from -0.242 to 0.376, indicating a nonsignificant difference between the two measures. Lastly, for the standard validity check question, the mean difference was 0.133 (SD = 0.776). The t-statistic was 0.941 (p = 0.354). The 95% confidence interval was from -0.156 to 0.423, demonstrating no significant difference in scores between the two measurements (Table 3).

**Table 3:** Paired t-test result of responses between the two measures p-value less than 0.05 is considered significant. M: mean, SD: standard deviation, SXI: Summated Xerostomia Inventory

Question	Difference		t-test	p-value	95% confidence interval	
	M	SD			Lower	Upper
SXI1	-0.167	0.834	-1.095	0.283	-0.478	0.145
SXI2	-0.233	0.971	-1.316	0.199	-0.596	0.129
SXI3	-0.233	0.858	-1.489	0.147	-0.554	0.087
SXI4	-0.167	0.648	-1.409	0.169	-0.409	0.075
SXI5	0.067	0.828	0.441	0.662	-0.242	0.376
Standard question	0.133	0.776	0.941	0.354	-0.156	0.423

In summary, none of the paired t-tests for the items in the Arabic SXI or the standard question reached statistical significance, indicating that there were no significant differences in the responses between the two measurement points. This supports the validity of the SXI as a reliable tool for repeated measures over time.

Summary of results

This study examined the reliability and validity of the Arabic SXI using a sample of 79 participants, of whom 57% were male and 43% were female. The participants’ ages ranged from five to 91 years with an average age of 40.76 years. They represented diverse demographic and health backgrounds, with the majority having benign pathology (73.4%) and a minority reporting to be smokers (13.9%). The SXI’s dependability was assessed using Cronbach’s alpha coefficients at two distinct instances. The initial assessment showed a Cronbach’s alpha of 0.824, implying a commendable internal consistency. The subsequent assessment presented an even superior alpha of 0.932, denoting outstanding internal consistency. The substantial Cronbach’s alpha values on both occasions reaffirm the Arabic SXI’s solidity and dependability. The Arabic SXI’s legitimacy was ascertained using a retest method and correlation analysis between the two measurements for every item. All SXI items displayed a potent positive correlation (between 0.746 and 0.871) with p-values below 0.001, exhibiting a remarkable consistency in responses over time. This uniformity is essential in verifying the SXI’s stability and authenticity. To check the disparity in responses between the two measurements, paired t-tests were carried out. The outcomes illustrated nonsignificant differences for all queries, suggesting that the responses were stable and not affected by changes in perception or occurrences.

## Discussion

The current study shows the process of creating an Arabic version of the SXI, which was administered to a cohort of well-defined head and neck patients who underwent parotidectomy in Saudi Arabia. The Arabic SXI demonstrated strong internal consistency, test-retest reliability, and construct validity. The XI questionnaire was originally used to evaluate the Austrian elderly [8]. It has since been translated and validated in different languages and for various populations [9,10-13]. The most recent translation was into an Indian language for head and neck patients who received radiation [14].

The mean total score of the Arabic SXI ranged from 10.40 to 11.69, which is comparable with other different populations’ mean scores. Scores have ranged from 6.50 in an Indonesian study [9] to 11.37 in a Chinese study [12]. In the current study, most of the patients underwent unilateral parotidectomy. The Cronbach’s alpha of the present study was .932, which denotes excellent internal consistency. This score was similar to that of the German SXI (α = 0.91) [11]. However, the score was higher than other validated studies in Chinese (α = 0.79) [12], Portuguese (α = 0.84) [13], Indonesian (α = 0.85) [9], and Indian (α = 0.81) [14].

We found that all SXI items showed a significant positive correlation (r = 0.746-0.871) with p-values less than 0.001, demonstrating exceptional stability in responses across time. This result was consistent with previous research conducted in Portuguese (r = 0.66) [13], Indonesian (r = 0.87) [9], and Indian (r = 0.79) [14] populations.

The present study showed a significant correlation (r = 0.766, p = 0.001) between the first and second measures similar to a previous validated study conducted in a Chinese population with an intraclass correlation coefficient of 0.8 [12]. In other validated studies reported in Portuguese [13], Indonesian [9], and Indian [14] populations, the intraclass correlation coefficient was 0.9.

The validity of a questionnaire refers to how accurately it gauges what it intends to measure. In this context, the validity of the questionnaire was determined by its ability to consistently measure responses over two instances. The paired-sample t-tests conducted in this study were aimed at identifying significant changes between two points of measurement. The absence of statistically significant differences indicates that the participants’ responses remained constant over time. This consistency suggests that one aspect of the questionnaire’s validity is its reliability and stability. Additionally, the nonsignificant differences imply that there were no significant shifts in perceptions or significant events that could have influenced the patients’ responses between the two measurements. This provides strong support for the questionnaire’s validity and its test-retest reliability.

The Arabic language is the sixth most spoken language. The Arabic SXI should be considered a helpful tool for clinical use in the Arabic-speaking population. Furthermore, the prevalence of xerostomia is nearly 100% in patients with Sjogren’s syndrome and those undergoing radiation therapy for head and neck malignancies [15]. Xerostomia is also common in young people using antidepressants, with a 22-fold increase in risk than people not taking antidepressants [16]. The Arabic translation and validation of the SXI questionnaire will be useful in Arabic patients with xerostomia. Additionally, in this study, phone calls were made to participants after the self-administration questionnaire to enhance participation and avoid cognitive burden bias [17]. Our research has some limitations. The population sample size was small, but it was equivalent to the average sample size of other international SXI validation studies. Additionally, the current study was only conducted in the Saudi population; other Arab countries were not represented among our participants.

## Conclusions

The research results confirm that the Arabic SXI possesses high dependability, as demonstrated by the potent Cronbach’s alpha values, and high legitimacy, as shown by the nonsignificant disparities between the test-retest measurements and the strong positive correlations. This implies that the Arabic SXI is a trustworthy and valid instrument for xerostomia measurement. The SXI is a short, reliable questionnaire that can be used to detect xerostomia more quickly and easily than other methods. It is important to translate the SXI into other languages and validate these versions to make sure that it can be used by patients from different cultural backgrounds and who speak other languages.

## Appendices

Supplementary data

Table 4 shows the Summated Xerostomia Inventory questionnaire.

**Table 4:** Summated Xerostomia Inventory questionnaire SXI: Summated Xerostomia Inventory, SXI-AR: Summated Xerostomia Inventory Arabic Version

	SXI	SXI-AR
SXI1	My mouth feels dry.	أشعر بالجفاف في فمي
SXI2	I have difficulty in eating dry foods.	أواجه صعوبة في تناول الأطعمة الجافة
SXI3	My mouth feels dry when eating a meal.	أشعر بالجفاف في فمي عند تناول الوجبات
SXI4	I have difficulties swallowing certain foods.	أواجه مشكلة في ابتلاع بعض أنواع الطعام
SXI5	My lips feel dry.	أشعر بجفاف في شفتاي
Scoring	1 = never, 2 = almost never, 3 = sometimes, 4 = often, 5 = always	١= لم تحصل ابداً، ٢= نادراً، ٣ = أحيانًا، ٤= كثيراً، ٥= دائمًا
